# Subclinical Cardiac Organ Damage in Patients with Moderate to Severe Psoriasis

**DOI:** 10.3390/jcm10112440

**Published:** 2021-05-31

**Authors:** Anja Linde, Eva Gerdts, Kåre Steinar Tveit, Ester Kringeland, Helga Midtbø

**Affiliations:** 1 Norwegian Research Centre for Women’s Health, Oslo University Hospital, 0424 Oslo, Norway; anja.linde@uib.no; 2 Department of Clinical Science, University of Bergen, 5020 Bergen, Norway; Eva.Gerdts@uib.no (E.G.); ester.kringeland@uib.no (E.K.); 3 Department of Dermatology, Haukeland University Hospital, 5021 Bergen, Norway; kare.steinar.tveit@helse-bergen.no; 4 Department of Heart Disease, Haukeland University Hospital, 5021 Bergen, Norway

**Keywords:** psoriasis, hypertension, subclinical cardiac organ damage, echocardiography

## Abstract

We explored the association between subclinical cardiac organ damage (OD) with comorbidities and psoriasis severity in 53 psoriasis patients on infliximab treatment (age 47 ± 15 years, 30% women) and 99 controls without psoriasis (age 47 ± 11 years, 28% women). Cardiac OD was assessed by echocardiography as the presence of increased left ventricular (LV) relative wall thickness (RWT), LV hypertrophy or dilated left atrium. Psoriasis severity was graded using the psoriasis area and severity index (PASI). The prevalence of hypertension was 66% in psoriasis vs. 61% in controls (*p* = 0.54) and cardiac OD seen in 51 and 73%, respectively (*p* = 0.007). Psoriasis was associated with a lower prevalence of cardiac OD (odds ratio (OR) 0.32, 95% confidence interval (CI) 0.13–0.77, *p* = 0.01) independent of age, sex, smoking, body mass index, and hypertension. Among psoriasis patients, hypertension was associated with increased risk of subclinical cardiac OD (OR 6.88, 95% CI 1.32–35.98, *p* = 0.02) independent of age, sex, and body mass index. PASI at treatment initiation was associated with a higher RWT at follow-up, independent of sex, age, and hypertension (β 0.36, *p* = 0.006) while no association with current PASI was found. In conclusion, cardiac OD was less prevalent in psoriasis patients on infliximab treatment than controls. Hypertension was the major covariable for subclinical cardiac OD in psoriasis.

## 1. Introduction

Psoriasis is a chronic, immune mediated, dermatological disease with a prevalence of 1.5–5% in Western countries [1]. The pathogenesis of psoriasis involves a complex interaction between keratinocytes, dendritic cells, and immune cells, as well as pro-inflammatory mediators such as tumour necrosis factor α [2]. Most psoriasis patients have a mild disease, but up to 15% have moderate to severe disease where a systemic treatment is indicated [3,4]. 

Having psoriasis has, in varying degree, been associated with an elevated risk of cardiovascular (CV) disease [5]. This is in part related to the high prevalence of CV risk factors in psoriasis, in particular hypertension and obesity [5,6]. In a British registry of patients with moderate to severe psoriasis receiving biological systemic treatment, 73% of patients were overweight or obese and 31% had hypertension [7]. In addition, psoriasis severity has been associated with both increased prevalence of CV risk factors and higher risk of CV disease [8]. Furthermore, the psoriasis treatment could influence CV risk. In a Danish retrospective longitudinal cohort study, patients on a biological systemic treatment had a 48% lower risk of CV death, myocardial infarction or stroke over 18 months of follow-up compared to patients on conventional therapies [9]. Clinical CV disease is preceded by structural and functional changes in the heart, collectively named subclinical cardiac organ damage [10]. Detection of subclinical cardiac organ damage with non-invasive CV imaging tests such as echocardiography is therefore recommended for early identification of subjects at particular high risk for CV disease, especially in hypertension [10,11]. In psoriasis, the prevalence of subclinical cardiac organ damage has been rarely studied, including in patients with moderate to severe psoriasis on biological systemic therapy. Thus, the aim of the current study was to assess if the prevalence of subclinical cardiac organ damage was increased in patients with moderate to severe psoriasis independent of CV risk factors compared to control subjects without psoriasis.

## 2. Materials and Methods

### 2.1. Study Population

Patients with moderate to severe psoriasis living on the west coast of Norway and receiving biological systemic treatment with infliximab at the Department of Dermatology, Haukeland University Hospital, Bergen, Norway were invited to participate in the study. The study exclusion criteria were known CV disease, defined as previous myocardial infarction, previous cardiac surgery and heart failure, current severe psychiatric disorders and expected low compliance. Fifty-five patients were screened of whom 53 patients fulfilled these criteria and were included in the study. Control subjects without psoriasis were recruited from the FAT associated CardiOvasculaR dysfunction (FATCOR) study, established at the Department of Heart Disease, Haukeland University Hospital. The FATCOR study has been described in detail previously [12]. In short, FATCOR is an observational, cross-sectional study of 620 women and men aged 30–65 years with a body mass index (BMI) >27.0 kg/m^2^ without known CV disease. Participants in FATCOR were examined between 2009 and 2016 with extensive CV imaging testing including echocardiography and ambulatory blood pressure (BP) monitoring. In total, 106 control subjects were included and matched for age, sex, and BMI with the psoriasis patients. Of these, seven control subjects had incomplete echocardiographic data and were excluded. Thus, the final study population consisted of 53 psoriasis patients and 99 control subjects. The study was approved by the Research Ethics Committees of Western Norway (REK 2016-02194) and conducted in accordance with the Declaration of Helsinki. All the study participants have signed a written informed consent.

### 2.2. Cardiovascular Risk Factor Assessment

The CV risk assessment was performed at the same time as the echocardiographic assessment. Participants filled in the self-reported medical history and medication in a standardized questionnaire. The information was quality assured against the hospital electronical medical records by the study physician. Office and 24-h ambulatory BP, height, body weight, and waist circumference were measured at the Department of Heart Disease by a trained study nurse. The office BP was measured in accordance with European guidelines on management of hypertension [10]. Elevated office BP was defined as BP ≥140/90 mmHg. Ambulatory 24-h BP was assessed using a Diasys Integra II device (Novacor, Cedex, France) with an appropriately sized cuff. The device was set to automatically measure BP and heart rate at 30-min intervals during night-time (11 p.m. and 7 a.m) and 15-min intervals during daytime. If <70% of the BP measurements were valid, the ambulatory recording was repeated. Elevated ambulatory BP was defined as average 24-h BP ≥130/80 mmHg [10]. Hypertension was considered present if the use of antihypertensive medication, history of hypertension or elevated ambulatory BP was present. Diabetes Mellitus was defined as elevated HbA_1c_ or a known history of diabetes.

### 2.3. Assessment of Quality of Life and Severity of Psoriasis

The severity of psoriasis was assessed by the psoriasis area and severity score (PASI) in accordance with European guidelines [4,13]. PASI quantitate the percentage of disease severity by grading skin involvement, skin erythema, induration, and desquamation and is currently the most used classification of psoriasis severity [4,13,14]. The dermatology life quality index (DLQI) was used to measure quality of life [4]. Information regarding DLQI and PASI at the start of biological systemic treatment and at the time of the echocardiographic assessment, was retrieved from the electronic medical records at the Department of Dermatology, Haukeland University Hospital. The C-reactive protein (CRP) was analyzed by Bevital AS by Matrix-Assisted Laser Desorption/Ionization Time-Of-Flight mass spectrometry (MALDI-TOF MS), as previously described [15].

### 2.4. Echocardiography

Echocardiography was performed using a Vivid E9 (GE Healthcare, Horten, Norway) scanner following a standardized protocol. Post-processing and analyses of digital echocardiographic images were performed at the Bergen Echocardiographic Core Laboratory, University of Bergen at workstations equipped with the Image Arena (TomTec, Unterschleissheim, Germany) software. Quantitative echocardiographic analyses were performed following the joint guidelines from the European Association of Cardiovascular Imaging and American Society of Echocardiography [16]. All images were first analyzed by a junior researcher and thereafter proof-read by a highly experienced researcher as recommended for echocardiography core laboratory operations [17]. The left ventricular (LV) function was assessed by biplane Simpson’s ejection fraction. LV hypertrophy was defined as the LV mass index >47.0 g/m^2.7^ in women and >50.0 g/m^2.7^ in men [10]. Concentric LV geometry was considered present if the relative wall thickness ≥0.43 [10]. Left atrial (LA) dilatation was defined as biplane LA end-systolic volume indexed for body height^2^ >18.5 mL/m^2^ in men and >16.5 mL/m^2^ in women [10,18]. Subclinical cardiac organ damage was considered present if concentric LV geometry, LV hypertrophy or dilated left atrium was found in the individual participant as recommended by guidelines [10].

### 2.5. Statistical Analysis

Statistical analyses and data management were performed using the IBM SPSS version 26 (IBM, Almonk, NY, USA). Continuous data are reported as the mean (standard deviation (SD)) for normally distributed variables or median (interquartile range) for non-normally distributed variables (CRP). Categorical variables are reported as numbers (percentages). Group comparisons were assessed by the two-sample Student’s *t*-test or Chi-square test as appropriate. Uni-and multivariable associations of subclinical cardiac organ damage were assessed by logistic regression analyses. The results were reported as odds ratios (OR) with corresponding 95% confidence intervals (CI). Univariable and multivariable associations of LA volume index, LV mass index, and LV relative wall thickness were assessed with linear regression analyses using the enter method and co-linearity tools. Results were reported as standardized β coefficients and *p*-values. Intra- and interobserver variability were assessed in 20 randomly selected patients by repeated analysis of LV mass. Reproducibility of LV mass was then assessed by the intraclass correlation coefficient (ICC) with 95% confidence intervals (CI). A two-tailed *p*-value of <0.05 was considered statistically significant in all analyses.

## 3. Results

### 3.1. Clinical Characteristics and CV Risk Factors

Hypertension was the most common CV risk factor, present in 66% of patients with psoriasis and 61% of control subjects, and 1/3 of participants were obese (Table 1). Smoking was more prevalent in psoriasis patients compared with controls (37 vs. 17%, *p* = 0.005) (Table 1).

The disease duration of psoriasis was long, with an average of 24 years. The mean PASI and DLQI at initiation of infliximab treatment indicated severe disease with a high impact on quality of life (Table 1). At the time of the present study, psoriasis patients had on average received infliximab treatment for 4.9 ± 3.8 years and PASI indicated very well treated psoriasis (Table 1). This was also reflected by the CRP levels that were similar between patients and controls (Table 1).

### 3.2. Prevalence and Covariables of Subclinical Cardiac Organ Damage

**Figure 1:**
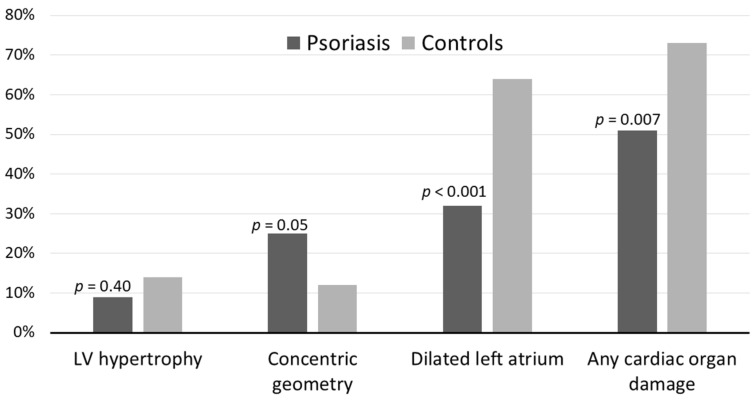
Subclinical cardiac organ damage in psoriasis patients and controls. LV: Left ventricle.

Psoriasis patients had a lower LV mass index and LA index, but a higher LV relative wall thickness compared to controls (*p* < 0.01) (Table 2). The prevalence of LV hypertrophy and concentric LV geometry were similar between groups, but the prevalence of LA dilatation was significantly lower in psoriasis patients (Figure 1). In the total study population, any subclinical cardiac organ damage was present in 51% of patients with psoriasis and 73% of control subjects (*p* = 0.007) (Table 2) (Figure 1). The reproducibility of LV mass was excellent with intra-observer variability (ICC 0.92, 95% CI 0.79–0.97) and interobserver variability (ICC 0.92, 95% CI 0.78–0.97).

In the total study cohort, the presence of subclinical cardiac organ damage was associated with female sex, presence of hypertension and older age, while having psoriasis was associated with a lower risk of subclinical cardiac organ damage in univariable logistic regression analyses (all *p* < 0.05) (Table 3). In multivariable analyses, having psoriasis remained associated with a lower prevalence of subclinical cardiac organ damage (OR 0.32, 95% CI 0.13–0.77, *p* = 0.01) independent of age, sex, smoking, BMI, and hypertension (Table 3). When looking at the different subcomponents of subclinical cardiac organ damage, having psoriasis was associated with a lower risk of LA dilatation in univariable analyses (OR 0.26, 95% CI 1.29–0.53, *p* < 0.001) and in multivariable analyses after adjusting for age, sex, smoking, hypertension, and BMI (OR 0.23, 95% CI 0.10–0.56, *p* = 0.001). Having psoriasis tended to be associated with an increase in LV concentric geometry in univariable analyses (OR 2.36, 95% CI 0.99–5.62, *p* = 0.05), but this association was further attenuated in multivariable analyses. Having psoriasis was not associated with LV hypertrophy.

Among psoriasis patients, the presence of subclinical organ damage was associated with higher age and hypertension in univariable logistic regression analyses (Table 4). In multivariable analyses, hypertension remained strongly associated with subclinical cardiac organ damage (OR 6.88, 95% CI 1.32-35.98, *p* = 0.02) after adjusting for age, sex, and BMI (Table 4).

PASI at the start of treatment or current PASI, were not associated with subclinical cardiac organ damage (Table 4). When looking at the different subcomponents of subclinical cardiac organ damage, PASI at the start of treatment was associated with the presence of concentric LV geometry (OR 1.08, 95% CI 1.00–1.16, *p* = 0.04), but this association was attenuated after adjusting for age, sex, and hypertension (OR 1.16, 95% CI 0.99–1.35, *p* = 0.06). When relative wall thickness as a continuous variable rather than a categorical (concentric LV geometry) variable was used, PASI at the start of treatment was associated with a higher LV relative wall thickness (β 0.35, *p* = 0.009), but not with LV mass index and LA volume index. In multivariable analyses, PASI at the start of treatment, remained associated with a higher relative wall thickness after adjusting for age, sex, and hypertension (β 0.36, *p* = 0.006). PASI at the start of treatment was not associated with LV hypertrophy or dilated LA. Actual PASI was not associated with LV concentric geometry, LA dilatation or LV hypertrophy.

## 4. Discussion

This study demonstrates that patients with psoriasis treated with infliximab have a lower prevalence of subclinical cardiac organ damage compared to control subjects without psoriasis, independent of high prevalence of CV risk factors such as hypertension, obesity, and smoking. Hypertension was the strongest correlate of subclinical cardiac organ damage in psoriasis patients, while a higher PASI at the start of infliximab treatment predicted the presence of concentric LV geometry even after 4.9 years of treatment.

Few previous studies have reported the prevalence and covariables of subclinical cardiac organ damage in psoriasis patients [19,20]. In our study, the prevalence of any type of cardiac organ damage was lower in psoriasis patients treated with infliximab compared to controls. This contrasts with a previous study by Atas et al. that reported higher left atrial volume in 40 psoriasis patients compared to healthy controls using three-dimensional echocardiography [19]. In another study of 216 psoriasis patients, Biyik et al. found that LV hypertrophy was present in 11% of psoriasis patients which was significantly higher than in controls [20]. In the current study, the prevalence of LV hypertrophy among psoriasis patients was 9%, which is similar to the prevalence reported by Biyik et al. However, both these previous studies included younger psoriasis patients with less severe comorbidities such as hypertension and obesity, shorter psoriasis disease duration, as well as less aggressive systemic treatment. Thus, the present results underscore the importance of CV risk factor management for the prevention of subclinical cardiac organ damage in psoriasis.

The present study adds to the current knowledge by demonstrating that psoriasis patients on an aggressive anti-inflammatory systemic treatment with infliximab and methotrexate had a lower prevalence of dilated LA and a comparable prevalence of LV hypertrophy to that found in controls. In psoriasis, the association of disease severity with subclinical cardiac organ damage has been rarely studied. However, previous research from other patient groups with chronic inflammation has indicated that the biological systemic treatment is associated with a lower prevalence of subclinical cardiac organ damage [21,22]. In 28 women with rheumatoid arthritis (RA), Daïen et al. found that the treatment with tumour necrosis factor alpha inhibitors (etanercept) reduced the LV mass index significantly after 6 months, independent of BP [21]. In another study of 23 women with RA, the 12-months treatment with infliximab was associated with a reduction in LV mass and N-terminal pro b-type natriuretic peptide [22]. On the other hand, a Dutch observational study of 51 RA patients receiving anti-tumour necrosis factor therapy (adalimumab, etanercept, certolizumab or golimumab) did not observe a change in LA volume or LV mass index during 6 months of treatment [23]. In the current study, psoriasis severity measured by PASI at the initiation of infliximab treatment was associated with a higher prevalence of concentric LV geometry 4.9 years later, but not with other types of subclinical cardiac organ damage. Concentric LV geometry has been particularly associated with an increased risk of myocardial infarction and sudden cardiac death in hypertension [24]. An association between disease activity and LV concentric geometry has been reported in RA patients previously [25,26]. Likewise, that systemic inflammation might mediate this process [27]. Taken together, we may speculate that earlier initiation of systemic biological treatment may protect better against adverse subclinical cardiac organ damage in psoriasis. However, further clinical studies are needed in this field.

In the present study, hypertension was the most prevalent CV risk factor in both psoriasis patients and the controls. Further, hypertension was the strongest covariable of subclinical cardiac organ damage in psoriasis patients. Hypertension and use of β-blockers have previously been associated with the development of psoriasis, and hypertension may therefore represent a common risk factor for both psoriasis and CV disease [28]. It is well known that untreated or longstanding hypertension causes subclinical cardiac organ damage such as LV hypertrophy, concentric LV geometry and, LA dilatation [10]. Hypertension has also been found to be an important driver of subclinical cardiac organ damage in other patient groups with inflammatory diseases [29]. In a study by Cioffi et al., who compared normotensive and hypertensive RA patients, a significantly higher prevalence of concentric LV geometry, LV hypertrophy, and a higher LA volume were found in RA patients with hypertension [30]. Together, these results indicate that hypertension detection and management is of particular importance for CV health in patients with inflammatory conditions such as psoriasis.

### Study Limitations

Our study has some limitations. Since the study has a cross-sectional design, causality between the factors and outcome cannot be established. Sex differences in subclinical cardiac organ damage are well described in hypertension and obesity [12,31,32], but the current study did not have sufficient statistical power to study sex-differences among psoriasis patients. Even though the incidence of psoriasis is equally distributed between sexes, men seem to more often receive biological systemic treatment compared to women [33,34,35], which is also reflected in this current study including only 30% women. The present study included only patients with moderate to severe psoriasis from one single geographical area. It is possible that the inclusion of patients with a greater variability in genetic background or range of psoriasis severity could have influenced the association between psoriasis severity and subclinical cardiac organ damage. Furthermore, since the study only included psoriasis patients receiving infliximab treatment, the effect of infliximab treatment on subclinical cardiac organ damage could not be assessed.

The strengths of this study include that we have used a core laboratory for the echocardiographic analysis, as recommended in the guidelines [17]. Furthermore, ambulatory BP measurements were used for an accurate diagnosis of sustained hypertension, and the homogenous group of psoriasis patients that were treated with infliximab, were matched to a control group without psoriasis but with a comparable CV risk factor burden.

## 5. Conclusions

In conclusion, patients with moderate to severe psoriasis had a lower prevalence of subclinical cardiac organ damage independent of CV risk factors compared to control subjects without psoriasis. Hypertension was the strongest covariable of subclinical cardiac organ damage in psoriasis patients. The current study underlines the importance of adequate hypertension management in clinical practice for psoriasis patients.

## Figures and Tables

**Table 1:** Clinical characteristics of psoriasis patients and controls.

	Psoriasis (*n* = 53)	Controls (*n* = 99)	*p-*Value
**Demographics**			
Age, years	47 (15)	47 (11)	0.94
Women, *n* (%)	16 (30)	28 (28)	0.81
Body mass index, kg/m²	29.2 (5.5)	29.9 (3.9)	0.42
Physical activity, hour/week	4.6 (3.7)	4.1 (3.4)	0.44
High-sensitive CRP, µg/mL, median (IQR)	1.0 (0.2–3.0)	1.1 (0.6–3.2)	0.33
Serum creatinine, µmol/L	75 (14)	77 (12)	0.54
Disease duration, years	24 (13)	-	
PASI at initiation of infliximab treatment	16.1 (11.5)	-	
PASI current	0.8 (0.8)	-	
DLQI at initiation of infliximab treatment	17.5 (6.0)	-	
DLQI current	0.7 (1.3)	-	
Psoriasis arthritis, *n* (%)	19 (36)	-	
**Cardiovascular risk factors**			
Current smoking, *n* (%)	19 (37)	15 (17)	0.005
Office systolic BP, mmHg	137 (17)	132 (17)	0.11
Office diastolic BP, mmHg	87 (8)	82 (9)	0.003
Ambulatory systolic BP, mmHg	123 (12)	122 (12)	0.75
Ambulatory diastolic BP, mmHg	80 (8)	79 (7)	0.60
Hypertension, *n* (%)	31 (66)	57 (61)	0.54
Obesity, *n* (%)	18 (34)	33 (33)	0.94
Diabetes, *n* (%)	3 (6)	5 (7)	0.87
LDL cholesterol, mmol/L	3.4 (0.9)	3.6 (0.9)	0.16
Statin, *n* (%)	6 (11)	11 (11)	1.00
**Medication**			
Treated hypertension, *n* (%)	15 (48)	20 (35)	0.22
Methotrexate, *n* (%)	45 (85)	-	
Infliximab, *n* (%)	53 (100)	-	
Duration of infliximab treatment, years	4.9 (3.8)	-	

BP: Blood pressure; CRP: C-reactive protein; DLQI: Dermatology life quality index; IQR: Interquartile range; LDL: Low density lipoprotein; PASI: Psoriasis area and severity index.

**Table 2:** Echocardiographic findings in psoriasis patients and controls.

	Psoriasis (*n* = 53)	Controls (*n* = 99)	*p-*Value
Interventricular septum thickness at end diastole, cm	1.0 (0.2)	1.1 (0.3)	0.007
LV diameter at end diastole, cm	4.8 (0.6)	5.1 (0.5)	0.005
LV posterior wall thickness at end diastole, cm	0.9 (0.2)	0.8 (0.2)	0.41
LV mass index, g/m^2.7^	36.1 (9.6)	40.3 (9.8)	0.01
LV hypertrophy, n (%)	5 (9)	14 (14)	0.40
LV relative wall thickness, ratio	0.38 (0.9)	0.34 (0.7)	0.001
Concentric geometry, n (%)	13 (25)	12 (12)	0.05
Ejection fraction, %	65 (6)	62 (5)	<0.001
Left atrial volume, mL/m^2^	15.9 (4.6)	19.3 (5.1)	<0.001
Dilated left atrium, n (%)	17 (32)	63 (64)	<0.001
Any cardiac organ damage, n (%)	27 (51)	72 (73)	0.007

LV: Left ventricle.

**Table 3:** Associations of subclinical cardiac organ damage in the total study cohort.

	Univariable Analyses		Multivariable Analyses	
	OR (95% CI)	*p*	OR (95% CI)	*p*
Psoriasis	0.39 (0.19–0.78)	0.008	0.32 (0.13–0.77)	0.01
Age, years	1.04 (1.01–1.08)	0.005	1.04 (1.00–1.07)	0.05
Female sex	2.67 (1.17–6.11)	0.02	5.15 (1.72–14.40)	0.003
Current smoking	0.33 (0.15–0.74)	0.006	0.51 (0.20–1.33)	0.17
Hypertension	2.21 (1.08–4.52)	0.03	1.81 (0.75–4.37)	0.19
Body mass index, kg/m^2^	1.07 (0.99–1.16)	0.10	1.06 (0.96–1.17)	0.22
Obesity	1.67 (0.80–3.46)	0.18		
Diabetes	4.29 (0.51–36.03)	0.18		
Anti-hypertensive treatment	2.57 (1.04–6.38)	0.04		
LDL cholesterol, mmol/L	1.28 (0.88–1.86)	0.20		
Statin, *n* (%)	1.99 (0.35–2.88)	1.00		

CI: Confidence interval; LDL: Low density lipoprotein; OR: Odds ratio.

**Table 4:** Associations of subclinical cardiac organ damage in the psoriasis group.

	Univariable Analyses		Multivariable Analyses	
	OR (95% CI)	*p*	OR (95% CI)	*p*
Age, years	1.05 (1.01–1.10)	0.02	1.05 (0.99–1.11)	0.12
Female sex	1.96 (0.59–6.52)	0.27	3.24 (0.66–15.86)	0.15
Hypertension	9.10 (2.11–39.34)	0.003	6.88 (1.32–35.98)	0.02
Body mass index, kg/m^2^	1.12 (1.00–1.25)	0.06	1.07 (0.93–1–24)	0.36
Current smoking	0.32 (0.10–1.05)	0.06		
Obesity	1.87 (0.59–5.94)	0.29		
Diabetes	2.08 (0.18–24.51)	0.56		
LDL cholesterol, mmol/L	1.81 (0.96–3.42)	0.07		
Statin, n (%)	2.01 (0.35–12.51)	0.42		
Anti-hypertensive treatment	5.87 (1.41–24.40)	0.02		
Methotrexate	1.01 (0.23–4.71)	0.95		
Disease duration, months	1.04 (0.99–1.08)	0.11		
PASI at treatment start	1.03 (0.97–1.08)	0.34		
Current PASI	1.75 (0.81–3.75)	0.15		
DLQI at treatment start	0.96 (0.86–1.06)	0.38		
Current DLQI	1.02 (0.68–1.54)	0.92		
Psoriasis arthritis	2.03 (0.79–5.27)	0.14		

CI: Confidence interval; DLQI: Dermatology life quality index; LDL: Low density lipoprotein; OR: Odds ratio; PASI: Psoriasis area and severity index.

## Data Availability

Data sharing not applicable.
